# Supplementary figures and images for: Parathyroid near-infrared autofluorescence differently benefits depending on the surgeon’s skill for preventing from hypoparathyroidism after total thyroidectomy: A systematic review and meta-analysis
Source: PLoS One. 2025 Apr 24;20(4):e0321310. doi: 10.1371/journal.pone.0321310 (PMC12021147; doi:10.1371/journal.pone.0321310)

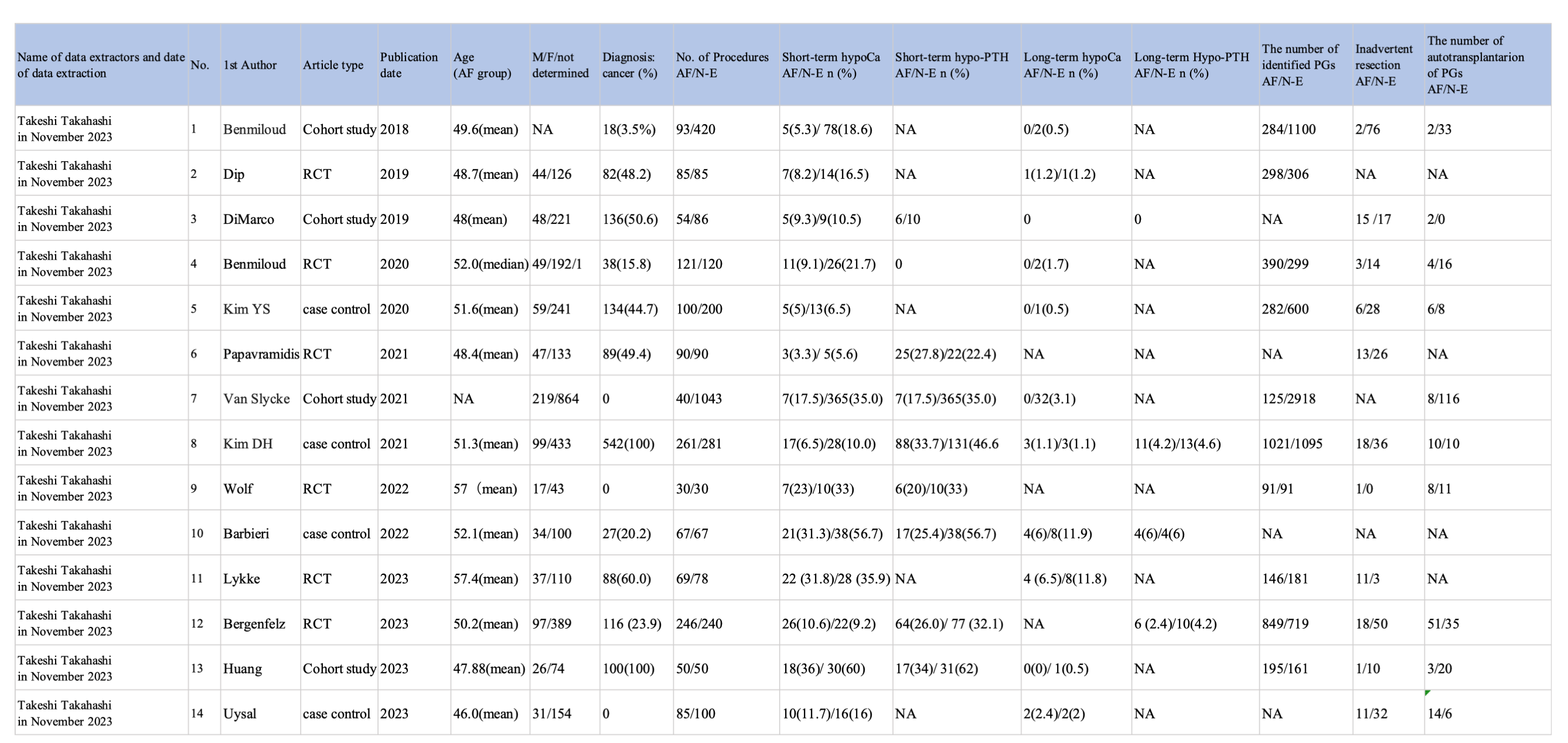

Supplement: S2 Table — (TIFF) [file pone.0321310.s002.tiff]
